# GENDER DIFFERENCES IN TYPES OF AGGRESSION USED AMONG DEMENTIA FAMILY CAREGIVERS

**DOI:** 10.1093/geroni/igae098.0498

**Published:** 2024-12-31

**Authors:** Wesley Browning, Mustafa Yildiz, Carolyn Pickering

**Affiliations:** The University of Texas Health Science Center Houston, Houston, Texas, United States; The University of Texas Health Houston, Houston, Texas, United States; The University of Texas Health Houston, Houston, Texas, United States

## Abstract

Investigations of family violence across the lifespan have revealed that men more often rely on physically aggressive tactics towards while women often rely on forms of psychological aggression. Physical aggression use also declines with age. We aimed to replicate, within a sample of dementia family caregivers, findings on gender, age and aggression from family violence research. We hypothesized that male family caregivers engage more often in physically aggressive behaviors towards their relative with dementia, while women engage more often in psychological aggression. Caregivers (n=453) self-reported frequency of neglect, psychological aggression, and physical aggression in the past 6-months at enrollment, 6-month and 12-month follow up. We utilized linear regression to investigate age and gender differences in abuse and neglect at baseline. We employed mixed models for repeated measures to investigate whether men engaged more often in abusive and neglectful behaviors across the three waves. Men (n=57) engaged more often in neglect and physical aggression than women (p’s<.05) at baseline. Caregivers who were older women engaged more often in neglect, while younger men engaged more often in physical abuse (p’s<.05). Based on our mixed model, men engaged more often in neglectful behaviors (B=-3.71, p=.001), physically aggressive behaviors (B=-1.55, p<.001) and overall total behaviors (B=-5.61, p=.001) than women across the three waves. Findings replicate those from prior studies examining family violence across the lifespan. Special attention should be paid to male caregivers in research and clinical practice as men are uniquely at risk for abusive and neglectful behaviors.

